# Supplementary material for: Localizing strain via micro-cage structure for stretchable pressure sensor arrays with ultralow spatial crosstalk
Source: Nat Commun. 2023 Mar 6;14:1252. doi: 10.1038/s41467-023-36885-3 (PMC9988987; doi:10.1038/s41467-023-36885-3)
Supplement: Supplementary file 1 — Supplementary Information [file 41467_2023_36885_MOESM1_ESM.pdf]

Supplementary Information

Localizing Strain *via* Micro-Cage Structure for Stretchable Pressure Sensor Arrays with Ultralow Spatial Crosstalk

Yufei Zhang[1, 2, 6], Qiuchun Lu[1, 3, 6], Jiang He[1, 4, 6], Zhihao Huo[1, 2], Runhui Zhou[1, 2], Xun Han[1], Mengmeng Jia[1, 2], Caofeng Pan[1, 2, 3], Zhong Lin Wang[1, 2, 5*], Junyi Zhai[1, 2, 3*]*

Table of Contents

Supplementary Notes 1

Supplementary Note 1: Preparation process and principle of the prslPDMS 1

Supplementary Note 2: Preparation process of the Ag NFs electrode 8

Supplementary Note 3: Preparation of graphene on microstructured PDMS film 11

Supplementary Note 4: Model analysis and simulation of micro-cage structure.......... 15

Supplementary Note 5: Principle and performance of pressure sensor 21

Supplementary Note 6: Pulse wave monitoring with ultrathin sensor........................... 26

Supplementary Note 7: Multiple sensors for grip detection 28

Supplementary Note 8: Principle and measurement system for sensor arrays 32

Supplementary References..40

Supplementary Notes

Supplementary Note 1: Preparation process and principle of the prslPDMS

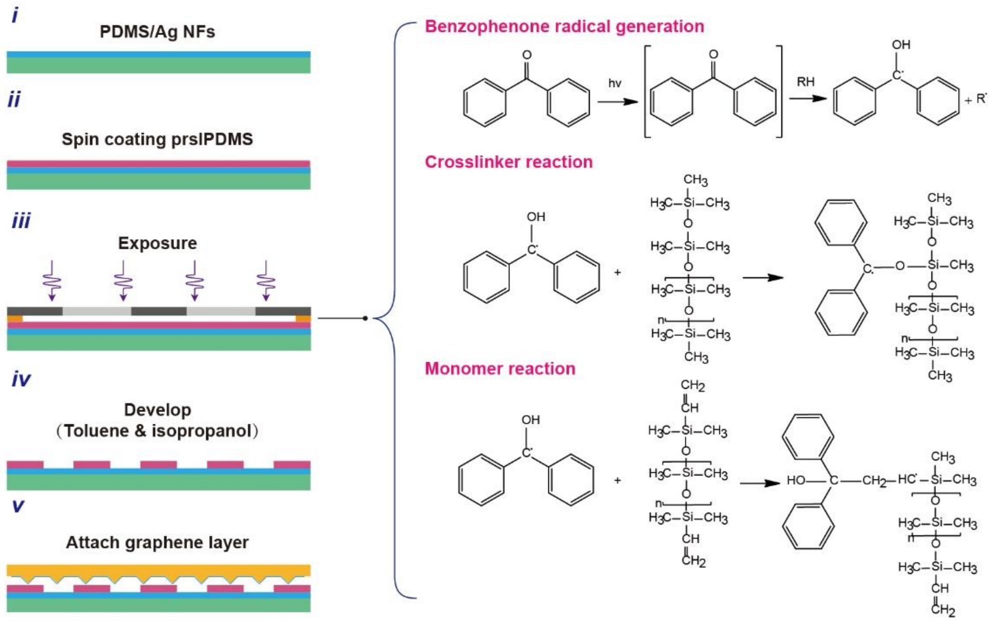

Supplementary Figure 1 | Preparation process and proposed chemistry of photo-reticulated strain localization PDMS.

The photo-reticulated strain localization PDMS (prslPDMS) was selected as the spacer layer due to its low cost, operation in ambient light and simple fabrication process. The conventional PDMS (SYLGARD184 Dow Corning) consists of the repeating -$OSi(CH_3)_2$- units. The PDMS base monomer is vinyl terminated, while the crosslinking monomers are methyl terminated which contain silicon hydride -$OSiHCH_3$- units. The PDMS monomers crosslink via a reaction between the monomer vinyl groups and the crosslinker silicon hydride groups to form Si-CH_2-CH_2-Si linkages during curing. However, the benzophenone radical is formed when it is irradiated of UV < 365 nm. And these radicals react with the silicon hydride groups presented in the PDMS crosslinkers and the vinyl groups of the PDMS monomers, which will prevent PDMS from undergoing the traditional crosslinking reactions. During the post exposure bake, the unexposed PDMS gets cured, while the exposed PDMS could be washed away in toluene. The detailed fabrication process is described below:

1. The PDMS was mixed thoroughly at a weight ratio of 1: 10 and stirred it to

obtain a homogeneous solution. Then, the benzophenone was dissolved in xylene at a weight ratio of 1: 10 and added to the conventional PDMS mixture to yield the prslPDMS solution with desired concentration.

2. The prslPDMS mixture was spin-coated on the desired substrate, and the thickness of the prslPDMS layer could be controlled by varying the spin speed.

3. The prslPDMS layer was selectively exposed to UV < 365 nm with the exposure power of 14 mW/cm^2 through a chrome mask for 10 min. Either a traditional lithography machine or a portable UV lamp could be used for exposure. A proximity exposure at a distance of ~ 100 um was used.

4. The samples should be put in a convection oven at 120 °C for approximately 150 s after the exposure processing. Put the sample in the toluene for 3 ~ 5 s to wash off the exposed regions and it will be rinsed with isopropanol and blown with N_2 gas. After the encapsulation of prslPDMS layer, the microstructured PDMS with graphene was laminated on its surface for sensor assembly.

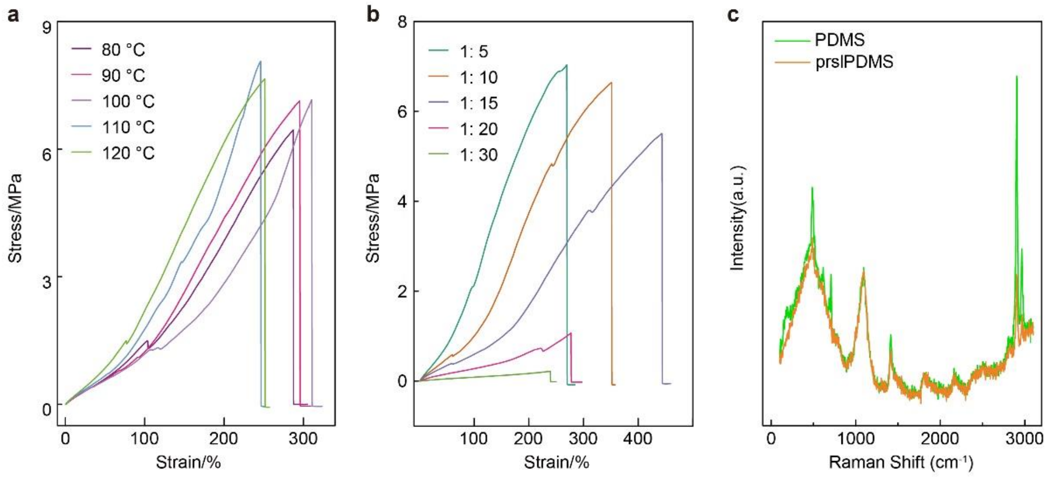

Supplementary Figure 2 | Tensile properties of PDMS. Tensile properties of PDMS with different curing temperature (**a**) and ratios (**b**). (**c**) Raman spectra of the PDMS and prslPDMS.

PDMS (SYLGARD184 Dow Corning) was mixed thoroughly at a weight ratio of 1: 10, but cured at different temperatures from 80 to 120 °C (Supplementary Fig. 2a). The result shows the temperature has little effect on its tensile properties since it has no obvious regularity. The elastic modulus of PDMS gradually decreases with the increasing proportion of base agent. The maximum tension first increases and then declines, reaching the maximum at the ratio of 1: 15, so the appropriate ratio could be selected to satisfy the practical application (Supplementary Fig. 2b). More detailed data could refer to the Supplementary Table 1. The Raman spectra of PDMS and prslPDMS demonstrate they have the similar peak locations (Supplementary Fig. 2c).

Supplementary Table 1 Tensile properties of PDMS with different ratios

	1: 5	1: 10	1: 15	1: 20	1: 30
Maximum Tension/%	269.3	350.9	443.5	277.1	239.1
Elastic Modulus/MPa (100%)	2.14	1.00	0.59	0.28	0.08
Maximum Elastic Modulus/MPa	7.03	6.64	5.51	1.06	0.22

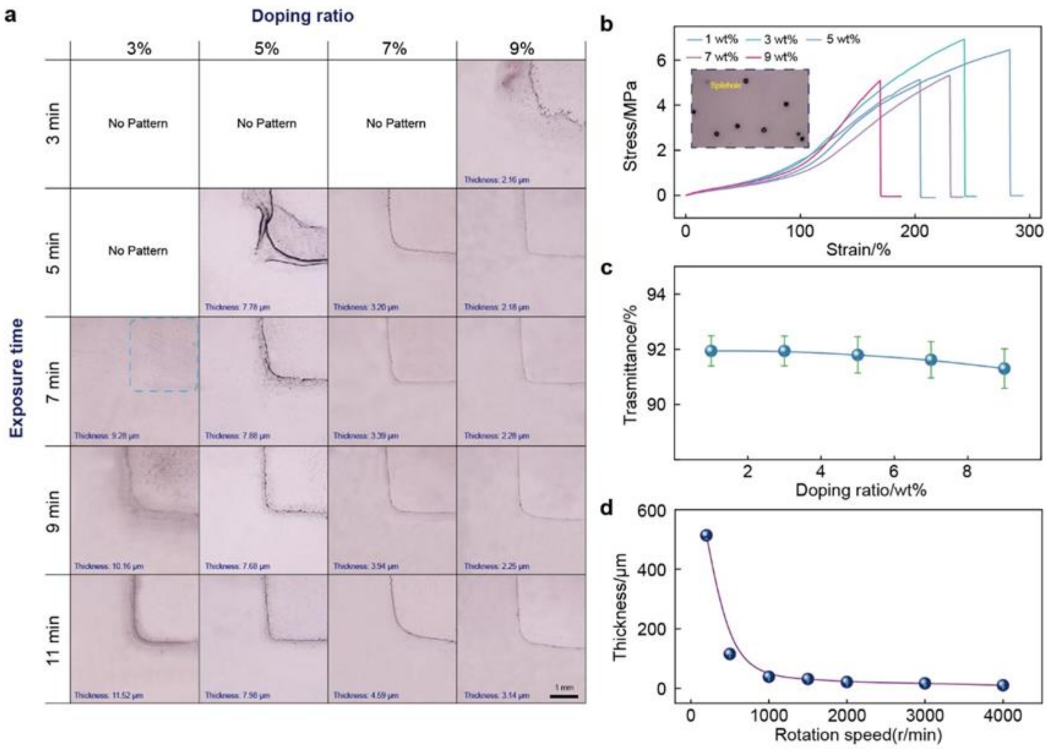

Supplementary Figure 3 | Effects of doping ratio and exposure time on prslPDMS patterning. (**a**) The optical photograph of patterned prslPDMS obtained under different benzophenone doping ratios and exposure time. Tensile properties (**b**) and transparency (**c**) of prslPDMS with different benzophenone doping ratios. (**d**) The thickness of the prslPDMS at various rotation speeds.

Supplementary Figure 3a shows the optical photograph of prslPDMS film obtained under different benzophenone doping ratios and exposure time. For the 9 wt% benzophenone doping, although the boundary is relatively rough, it only takes 3 min of exposure to generate the desired pattern. However, for the 3 wt% benzophenone doping, there are faint pattern traces when the exposure time increases to 7 min. The required pattern won't produce until the exposure time reaches to 11 min. Therefore, it could be inferred that the high proportion of benzophenone will produce the desired pattern at a shorter exposure time, which may be attributed to the fact that the more benzophenone radicals effectively inhibit the PDMS crosslinking. Furthermore, the tensile properties of PDMS gradually decline with the increasing benzophenone doping ratios (Supplementary Fig. 3b). Benzophenone cannot be completely dissolved in xylene with the high doping ratio, and many particles generate during the preparation process which

will further turn into spileholes mixed in PDMS film, thus reducing the tensile properties of PDMS. Meanwhile, the transparency of the prslPDMS film also slowly decreases from 91.93 % to 91.29 % (Supplementary Fig. 2c), and the thickness of the PDMS film could be controlled by varying the rotation speed from 514.04 μm to 2.53 μm (Supplementary Fig. 3d). Hence, the prslPDMS film is suitable for the applications in transparent stretchable devices.

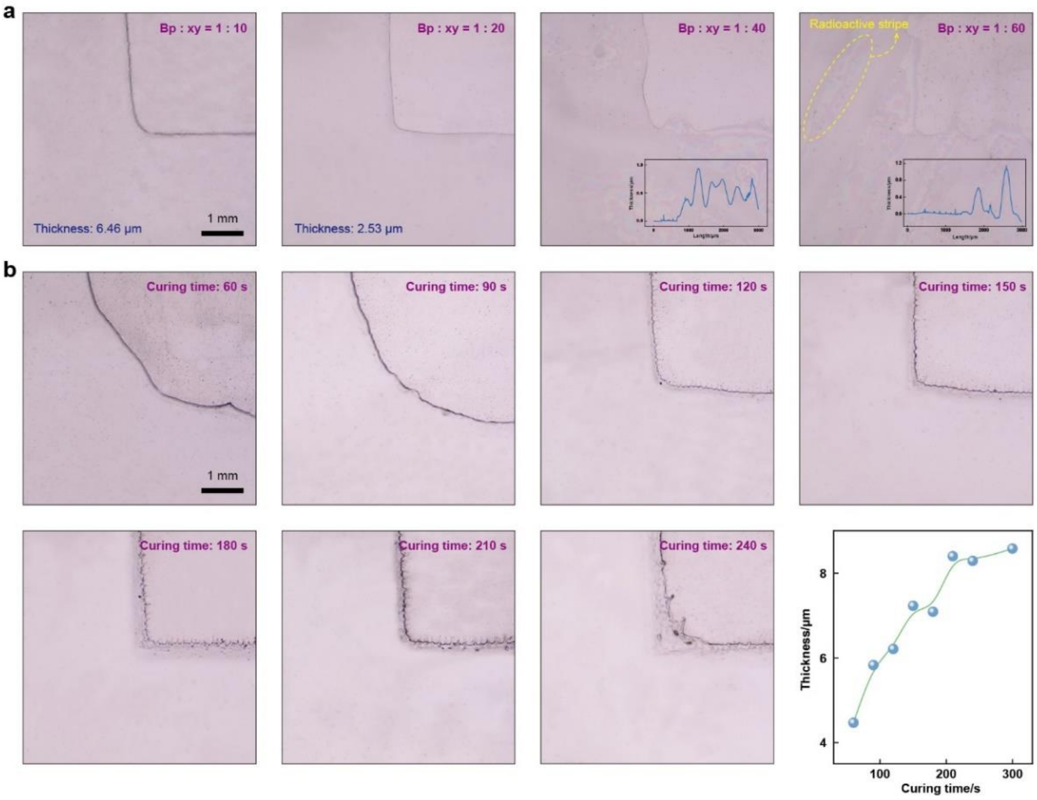

Supplementary Figure 4 | Effects of xylene content and curing time on prslPDMS patterning. Optical photograph of patterned prslPDMS with different xylene contents (**a**) and curing time (**b**).

The role of xylene in the prslPDMS solution is similar to the diluent, and the thinner and higher precision patterned films could be prepared by increasing the content of xylene. However, when the ratio of benzophenone to xylene exceeds 1: 40, the PDMS cannot be spin-coated on the substrate surface smoothly. And there are many radial stripes, which is not conducive to the preparation of the whole devices (Supplementary Fig. 4a). Supplementary Figure 4b shows that a shorter curing time will make the unexposed area not fully cured, resulting in a larger patterned area. Nevertheless, a longer curing time will also cause the exposed areas to cure, and the boundaries will become rougher. The results also reveal that the thicker prslPDMS film could be obtained with the increasing curing time. Therefore, a proper curing time is crucial for the preparation of prslPDMS films.

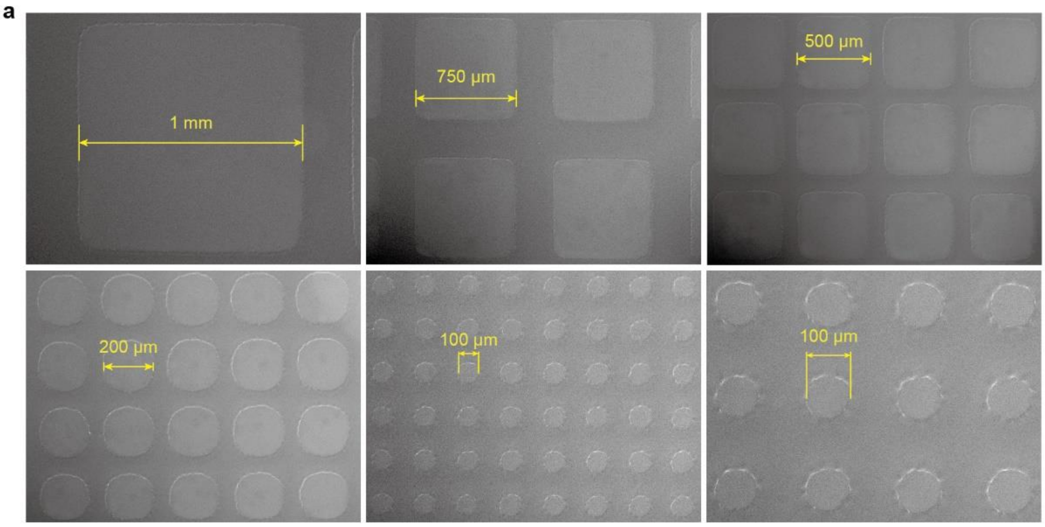

Supplementary Figure 5 | The SEM images of prslPDMS with different resolutions.

Supplementary Figure 5 shows the patterned prslPDMS film with different precisions ranging from 1 mm to 100 μm. The prslPDMS film gradually changes from square holes to round holes with the increasing exposure resolution. In addition, it was found that the accuracy of 50 μm could be achieved, but its uniformity is poor, so we believe that the resolution of prslPDMS film could reach 100 μm.

Supplementary Note 2: Preparation process of the Ag NFs electrode

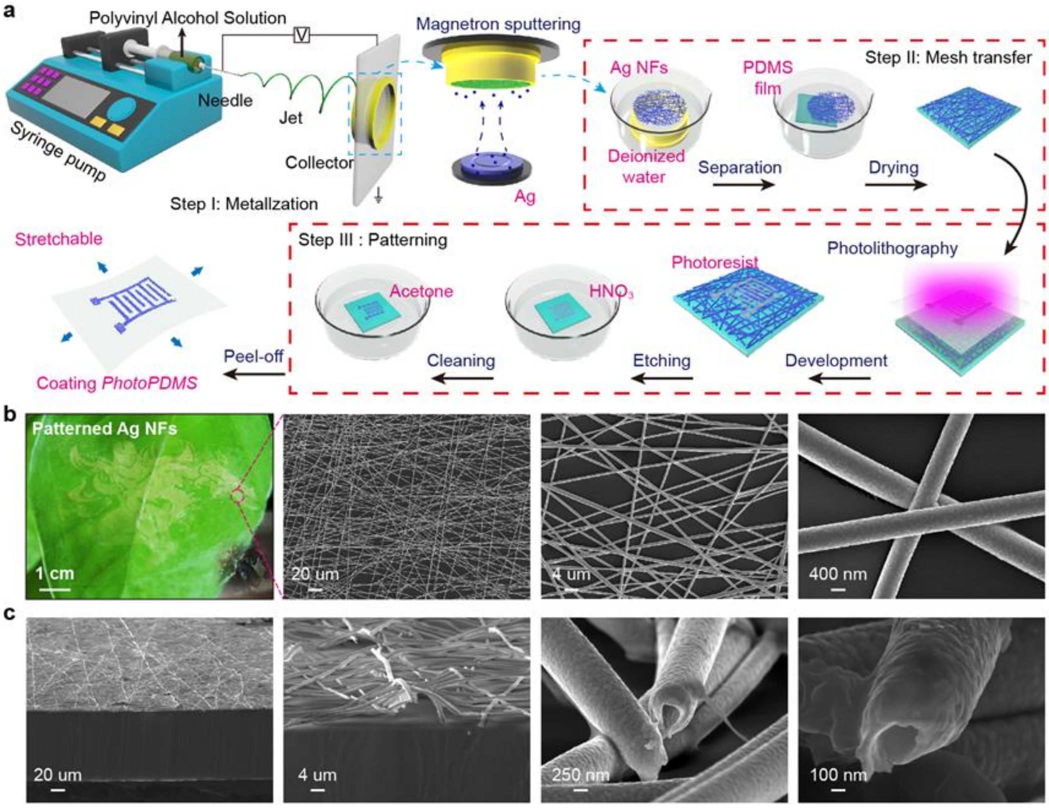

Supplementary Figure 6 | Fabrication process of the patterned Ag NFs electrode. (**a**) Schematic of preparation process for the Ag NFs interdigital electrodes. (**b**) Optical photograph of the large-area patterned Ag NFs electrode on PDMS substrate and SEM images of Ag NFs under different magnifications. (**c**) Cross-sectional SEM images of the Ag NFs.

The procedure to prepare the patterned Ag NFs electrode is described below:

1. Electrospinning: Polyvinyl alcohol water solution with a concentration of 10 wt% was prepared by mixing PVA powder (molecular weight, 80000) with deionized water. The mixture was stirred at 700 r/min at a temperature of 40 °C for 48 h by using a magnetic stirrer to obtain a homogeneous solution. Then, the PVA aqueous solution was loaded into a 10 ml plastic syringe with a blunted G10 needle for electrospinning. The air humidity should be maintained at around 25%. The flow rate of the solution in the syringe pump was controlled at 0.4 ml/h, and a constant potential of 13 kV (negative voltage of 4kV and positive voltage of 9kV) was employed between the needle and the grounded substrates (acrylic frame covered with aluminum foil) with a distance of 10

cm. The random PVA NFs could be obtained by using the circular collectors with an average diameter of 450 nm.

2. Metallization: The random PVA NFs was then coated with a thin layer of silver by magnetron sputtering. Typically, the power was 100 W and the sputtering time was 15 min, which will provide a uniform conformal coating on the PVA NFs to form a concave or core-shell Ag NFs. The average diameter of these Ag NFs was about 700 nm.

3. NFs transfer process: Firstly, PDMS (SYLGARD184 Dow Corning) was mixed thoroughly at a weight ratio of 1: 10 and degassed for 5 min to remove air bubbles. It was then spin-coated onto a glass substrate and cured in the oven (120 °C, 20 min). Next, put the fabricated Ag NFs into the water, and were then transferred to the PDMS substrate at a certain angle. The surface of the Ag NFs film was slowly dried with a heat blower. After the film was dried, put it into the water and dried again (repeat three times). Multiple evaporation of the water will fully enhance the adhesion between the Ag NFs and PDMS substrate. Subsequently, the Ag NFs film was put into an oven at 180 °C and annealed for 20 minutes. It will eliminate the internal stress of Ag NFs and strengthen the crosslinking among the fibers, which will improve its electrical conductivity and tensile properties. Finally, a drop of methanol was added to enhance the physical adhesion between the Ag NFs and the substrate surface.

4. Photolithography: The positive photoresist (AZ5214) was spin-coated on the surface of the Ag NFs at 1000 rpm for 60s and dried it on the hot plate with the temperature of 100°C. The exposure power is 15 mW/cm^2 and the exposure time is about 20 s. After exposure and development, the remaining photoresist as a mask layer would protect the Ag NFs from chemical etching.

5. Etching: The uncovered Ag NFs will be etched by the 5 M dilute nitrate solution in 30 seconds. Shake gently during etching to remove the bubbles and use acetone to clean the sample several times to remove the residual photoresist. The remaining Ag NFs are the desired patterned electrode.

The fabrication process of the transparent stretchable electrodes with Ag NFs in the elastomeric polydimethylsiloxane (PDMS) array are shown in the Supplementary

Figure 6a. A digital photograph of cartoon figure based on the Ag NFs is demonstrated, which exhibits the high precision, transparency, and conformal adhesion on the irregular surface. The SEM images show random distribution of continuous nanofibers network, and its diameter is about 700 nm. The cross-sectional images of the Ag NFs (Supplementary Figure 6c) confirm that PVA NFs templates have been fully dissolved, resulting in a concave or core/shell structure.

Supplementary Note 3: Preparation of graphene on microstructured PDMS film

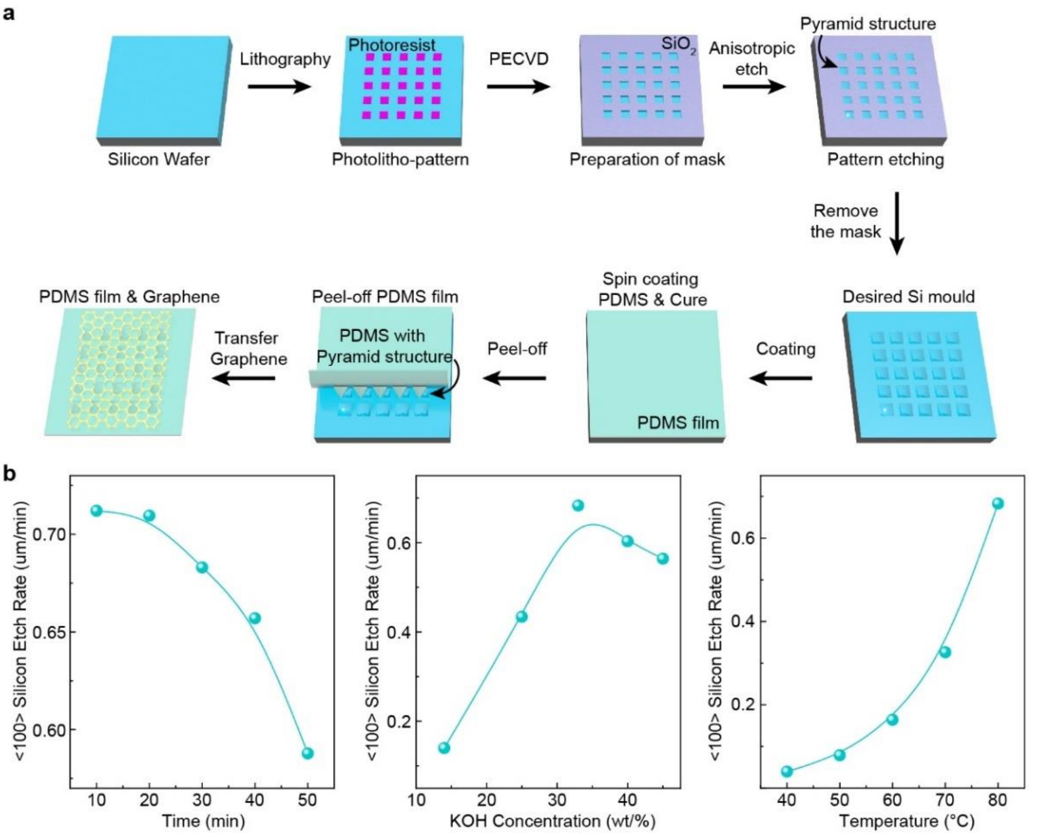

Supplementary Figure 7 | The preparation process of the silicon template by wet etching. (**a**) Schematic illustration of the fabrication sequence of the silicon template and microstructured PDMS film. (**b**) The influence of the etching time, KOH concentration and etching temperature to the etching rate of silicon, respectively.

The detailed preparation process of the silicon template is shown in the Supplementary Figure 7a.

1. A uniform standard <100> n-type silicon wafer was used as the starting material. It is essential to treat the silicon surface by using the plasma cleaner (O_2, 100 sccm, 100 W, 30 min) to enhance the adhesion between the silicon substrate and photoresist.

2. The positive photoresist (S1813) was spin-coated on the surface of the silicon wafer, and the patterned photoresist masks could be obtained by using the traditional UV photolithography technique.

3. The SiO_2 layer was sequentially deposited on the top of the silicon wafer, and the thickness was about 700 μm via plasma enhanced chemical vapor deposition

(PECVD). The silicon wafer was then ultrasonically treated in acetone for 2 h to remove the photoresist, and the remaining SiO_2 layer could prevent the Si substrate from etching in the KOH solution.

4. The anisotropic etching was carried out when put the silicon wafer in the pre-configured etching solution, which is composed of the KOH and isopropanol. Subsequently, we have studied the influence of the etching time, KOH concentration and etching temperature to the etching rate of the silicon in order to obtain the optimal silicon template, as illustrated in Supplementary Figure 7b. The results show that the etching rate of <100> silicon crystal plane decreases with the etching time, but it is positively correlated with the etching temperature. As for the KOH concentration, the etching rate first increases with its concentration, then gradually decreases, which indicates the optimal KOH concentration is about 33%.

5. The silicon template should be treated with the trimethylchlorosilane (TMCS) to prevent PDMS from sticking on the surface of the silicon template. A small amount of TMCS is added to the surface of the silicon template and dried it with a heat blower. The PDMS was mixed thoroughly at a weight ratio of 1: 10 and degassed by using vacuum drying chamber. Pour the PDMS mixture over the silicon template and rotate at a certain speed. The sample should be degassed again to remove the remaining air bubbles. Then, put the sample into an oven for 20 min at 120 °C and the microstructured PDMS could be easily peeled-off after curing.

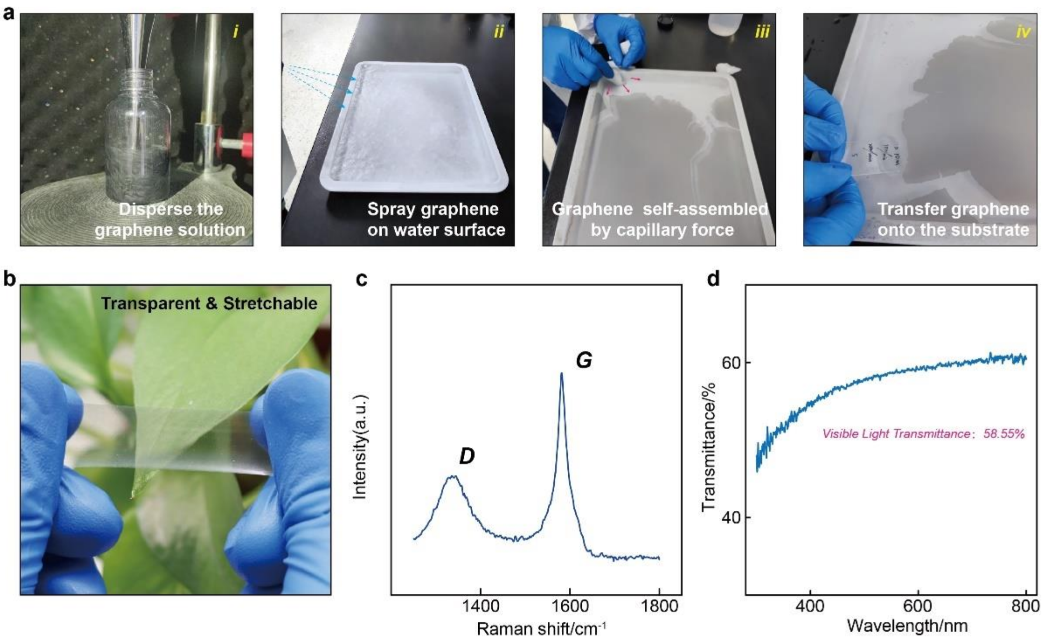

Supplementary Figure 8 | The preparation process of the graphene film. (**a**) Schematic illustration of the fabrication sequence of the self-assembled graphene film. (**b**) Optical photograph of graphene films attached on the PDMS substrate. Raman spectra (**c**) and UV-visible spectra (**d**) of the Gr/PDMS films.

The detailed fabrication process of the self-assembled graphene film is shown in the Supplementary Figure 8a. The graphene sheets were first dispersed in anhydrous ethanol solution, followed by strong ultrasonication for 1 h to obtain a stable dispersion with appropriate aging time. The ethanol-assisted graphene dispersion solution was sprayed on the water surface, leading to the formation of uniform Langmuir monolayers at the liquid/air interface. Then, the sponges were put on one side of the interface to quickly siphon water from the system, and the homogeneous Langmuir monolayers were closely squeezed in the opposite direction to the siphon. When the film stopped moving and the siphon effect failed to drive the film, a self-assembled graphene film was ultimately prepared on the water surface. Finally, the graphene films were transferred on the microstructured PDMS film, and it would be dried by hot wind for 3 min to improve their stability. The graphene films possess the good transparency and stretchability (Supplementary Fig. 8b), and its average transmittance in the visible light is 58.55 % (Supplementary Fig. 8d). Raman spectrum of graphene shows a small I_D/I_G (~ 0.84) ratio, indicating the high quality of graphene films (Supplementary Fig. 8c).

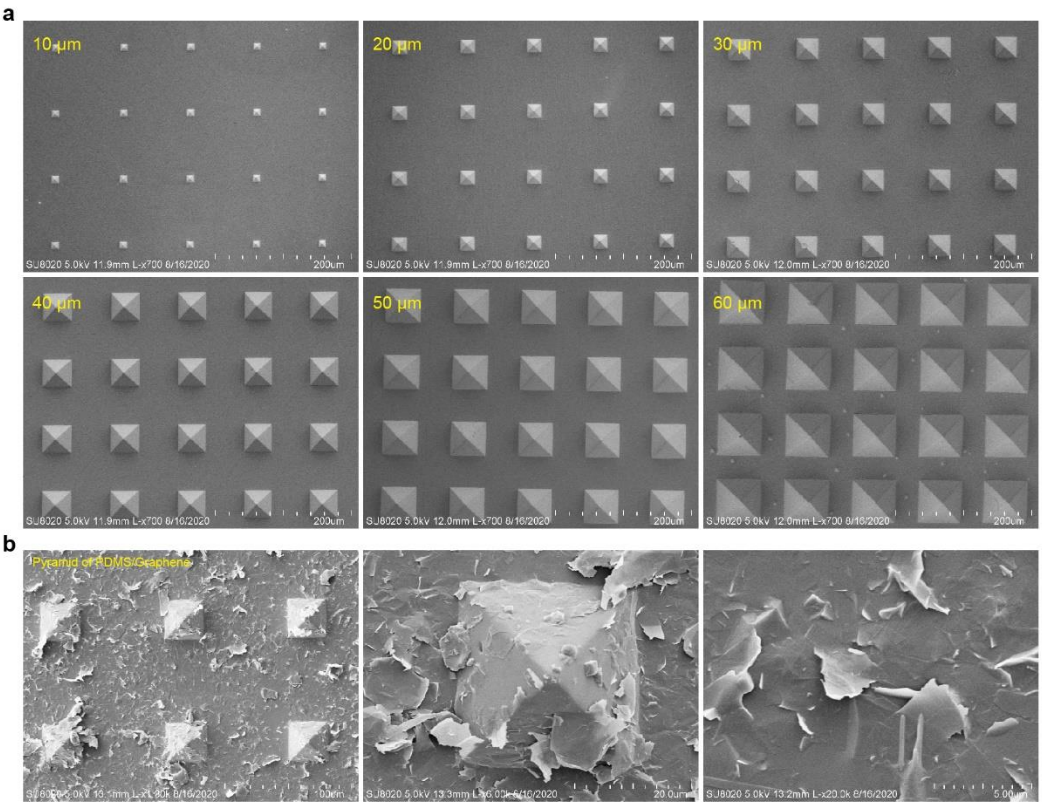

Supplementary Figure 9 | SEM images of the microstructured PDMS and graphene. (**a**) SEM images of the patterned PDMS with various pyramid sizes. (**b**) SEM images of the graphene transferred to the patterned PDMS.

According to the method mentioned above, the size of pyramidal microstructure could be controlled by preparing a variety of silicon templates ranging from 10 μm to 60 μm (Supplementary Fig. 9a). Another layer of graphene will be transferred on the surface of the microstructured PDMS, and the corresponding SEM images are shown in Supplementary Figure 9b.

Supplementary Note 4: Model analysis and simulation of micro-cage structure

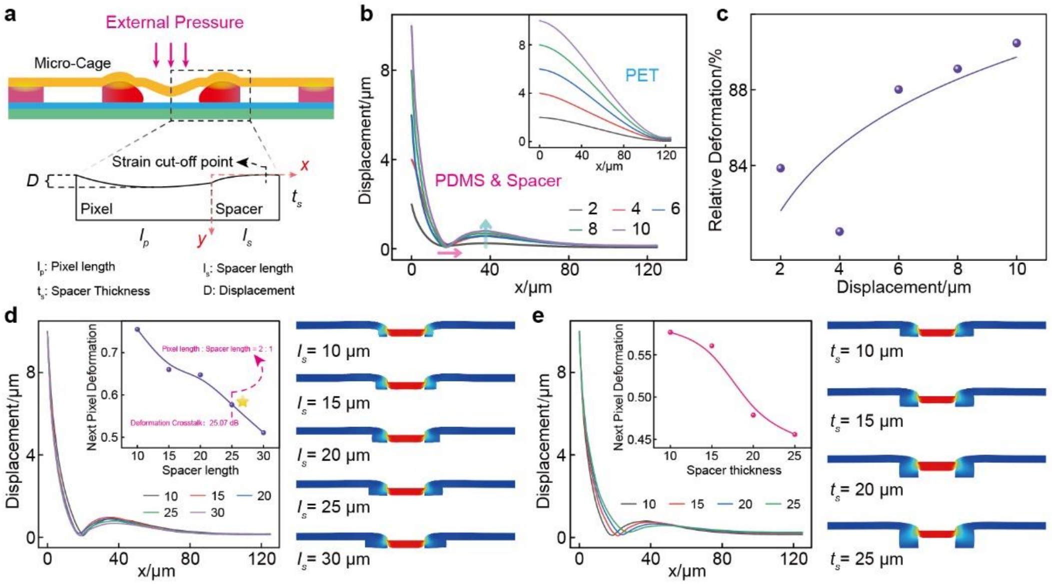

Supplementary Figure 10 | Two-dimensional simulation analysis of the boundary of micro-cage structure. (**a**) Simple geometric model of the pressure sensor under external pressure. (**b**) Deformation along the x-axis of the pressure sensors based on PET or PDMS & Spacer under different external displacements, respectively. (**c**) The relative deformation of adjacent pixel compared to the stressed pixel under different external displacements. Deformation along the x-axis of the pressure sensors based on the PDMS & Spacer with the different spacer lengths (**d**) and spacer thicknesses (**e**).

A simple two-dimensional geometric model is proposed to analyze the device deformation under external displacements, as shown in Supplementary Fig. 10a. The prslPDMS spacer layer is crucial for the pressure sensor, which could not only adhere the top and bottom electrodes, but also form a micro-cage structure to isolate the mechanical crosstalk. It could be found that the whole model has four key parameters, which are pixel length (l_p), spacer length (l_s) and thickness (t_s), and external displacement (D). When a pixel is subjected to external pressure, the top electrode will also deform due to its good elasticity, thereby gradually expanding to adjacent pixels, resulting in mechanical crosstalk. Therefore, we establish a coordinate system on the right boundary of the pixel to observe the elongation strain along the x-axis and y-axis. Supplementary Figure 10b shows the deformation along the x-axis of the model based

on the PDMS & Spacer with the displacements ranging from 2 μm to 10 μm, and the inset in the upper right corner is the PET deformation with the same conditions (l_p = 50 μm, l_s = 25 μm, t_s = 10 μm). The results show that the strain along the x-axis of the model using PDMS & Spacer layer drops rapidly to a small value, which is called the strain cut-off point. Owing to the warping of the PDMS, there will also be an increased displacement in some regions after the cut-off point, but the overall value is still in a small range. Conversely, for the PET model, the deformation along the x-axis decreases slowly, which means the adjacent pixels must be subjected to large mechanical crosstalk. Then, we quantitatively analyze the relative deformation of adjacent pixel with the stressed pixel under different displacements (Supplementary Fig. 10c). It could be seen that the relative deformation also gradually increases with the external displacements, indicating that the prslPDMS spacer layer could effectively isolate the crosstalk

Supplementary Figure 10d and 10e respectively depict the effect of the spacer length and thickness on the isolation of crosstalk. The increase of the spacer length has no obvious effect on the strain cut-off point, but the displacement of the adjacent pixel drops significantly (l_p = 50 μm, t_s = 10 μm, D = 10 μm). Additionally, the calculation formula of mechanical crosstalk isolation is as follows.

$$I_{so} = -20\log\frac{D_{adjacent\ \ pixel}}{D_{stressed\ \ pixel}} \tag{4-1}$$

The crosstalk isolation (I_{so}) is 22.43 dB when the spacer length is 10 μm, but it becomes 25.83 dB with the spacer length increases to 30 μm, indicating that the longer spacer length is more effective to avoid crosstalk. Nevertheless, an excessively long spacer is not conducive to design the array device, so it is considered that the I_{so} above 25 dB has sufficient crosstalk isolation effect, and the ratio of spacer length to pixel length at this time is 1: 2. More detailed comparison data could refer to Supplementary Table 2 and Supplementary Table 3. In practical applications, if the higher density devices need to be prepared and crosstalk isolation requirements are not very strict, this ratio could also be appropriately increased, such as 1: 5, or even 1: 10, etc. The effect of spacer thickness on crosstalk isolation is similar to that of external displacements, and the thicker spacer layer means the smaller relative deformation with the same external displacement.

Therefore, the simulation results show that although the strain cut-off point tends to shift to right, the deformation of adjacent pixels will decline with the increase of the spacer thickness. Moreover, the thickness of spacer layer will affect the detection range of the pressure sensor, so its thickness needs to be selected according to the actual situation.

Supplementary Table 2 Crosstalk isolation between PET and prslPDMS

	Displacement/μm	Displacement of adjacent pixel/μm	Crosstalk isolation/dB
PDMS & prslPDMS	2	0.19494	20.22258
	4	0.45338	18.91195
	6	0.43939	22.70602
	8	0.52455	23.66606
	10	0.58465	24.66208
PET	2	1.20711	4.38566
	4	2.3246	4.71424
	6	3.66558	4.28017
	8	4.81495	4.40996
	10	6.13427	4.24474

Supplementary Table 3 Crosstalk isolation of prslPDMS at different parameters

l_P: l_S: t_S/μm	Displacement/μm	Displacement of adjacent pixel/μm	Crosstalk isolation/dB
50: 10: 10	Max (10)	0.75512	22.43968
50: 15: 10		0.66014	23.60728
50: 20: 10		0.64746	23.77574
50: 25: 10		0.55772	25.07168
50: 30: 10		0.51086	25.83396
50: 25: 15	10	0.75512	25.02554
50: 25: 20	10	0.66014	26.39964
50: 25: 25	10	0.64746	26.82432

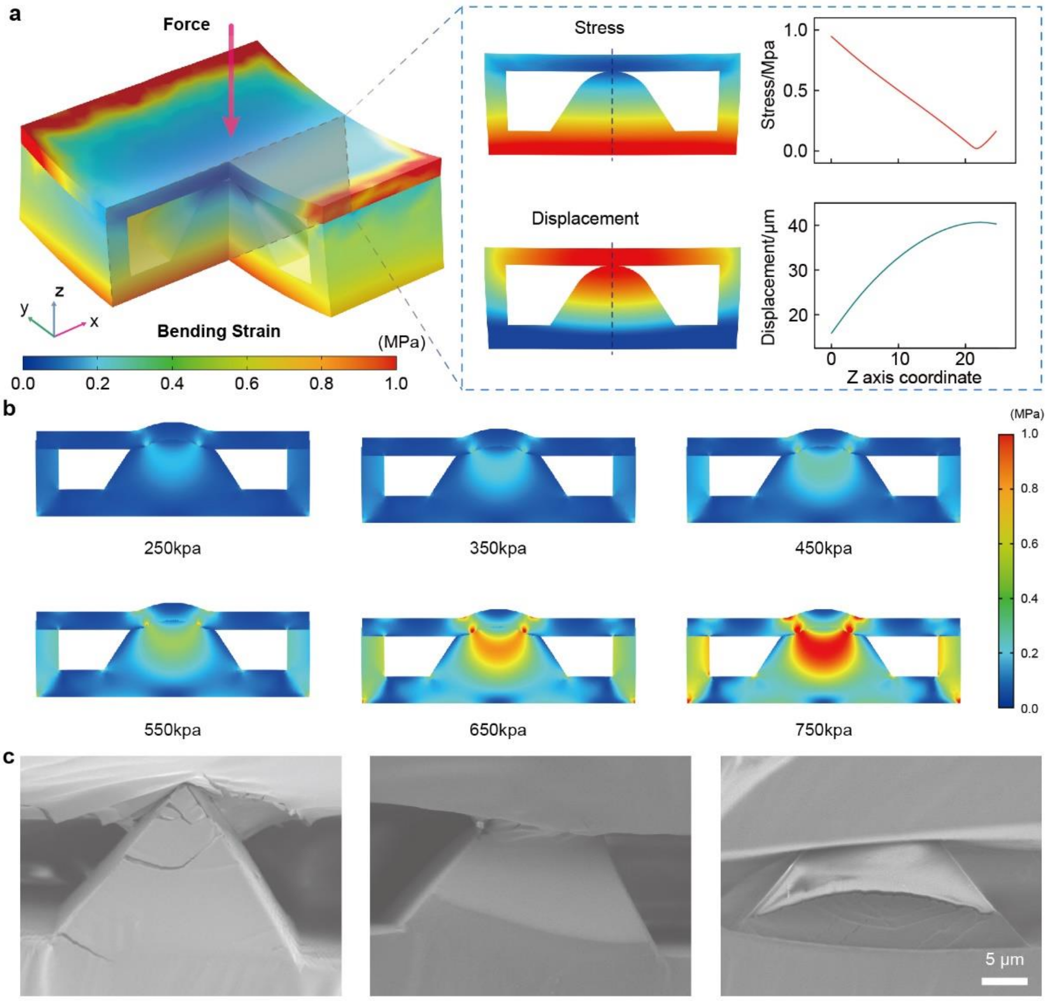

Supplementary Figure 11 | Simulation analysis inside the micro-cage structure. (**a**) 3D simulation of the micro-cage structure subjected to both bending strain and external pressure. Inset: Stress distribution and displacement in the xz plane, and the data on the central line along the z-axis in shown on the right. (**b**) Finite element simulation of microstructures at different elastic modulus. (**c**) SEM images of microstructures under different pressures.

The micro-cage structure model of the pressure sensor is calculated based on the solid mechanics modulus in COMSOL Multiphysics, and this model involves the top electrode laminated on the microstructured PDMS layer (Supplementary Fig. 11a). The entire micro-cage structure is encapsulated in PDMS, which imposes fixed constraint on one end, a prescribed displacement on the other end, and roller support on its bottom surface, thereby exerting a bending effect on the above model. Moreover, the displacements are applied to the upper surface of the device and the Von Mises Stress

yield criterion is used to observe the stress distribution of sensors on its xz plane. Bending strain leads to the large stress in the bottom, while the upper surface also has stress concentration due to the external pressure, and stress data on the central line along the z-axis is displayed on the right. Meanwhile, the displacement of the bottom is small due to the fixed constraint, but the displacement of the upper surface can reach 40 μm which could make stacked structure in close contact. Supplementary Figure11b shows the stress concentration on pyramid is obviously enhanced with the external pressure, which is confirmed by the progressively larger cracks in SEM images (Supplementary Fig. 11c).

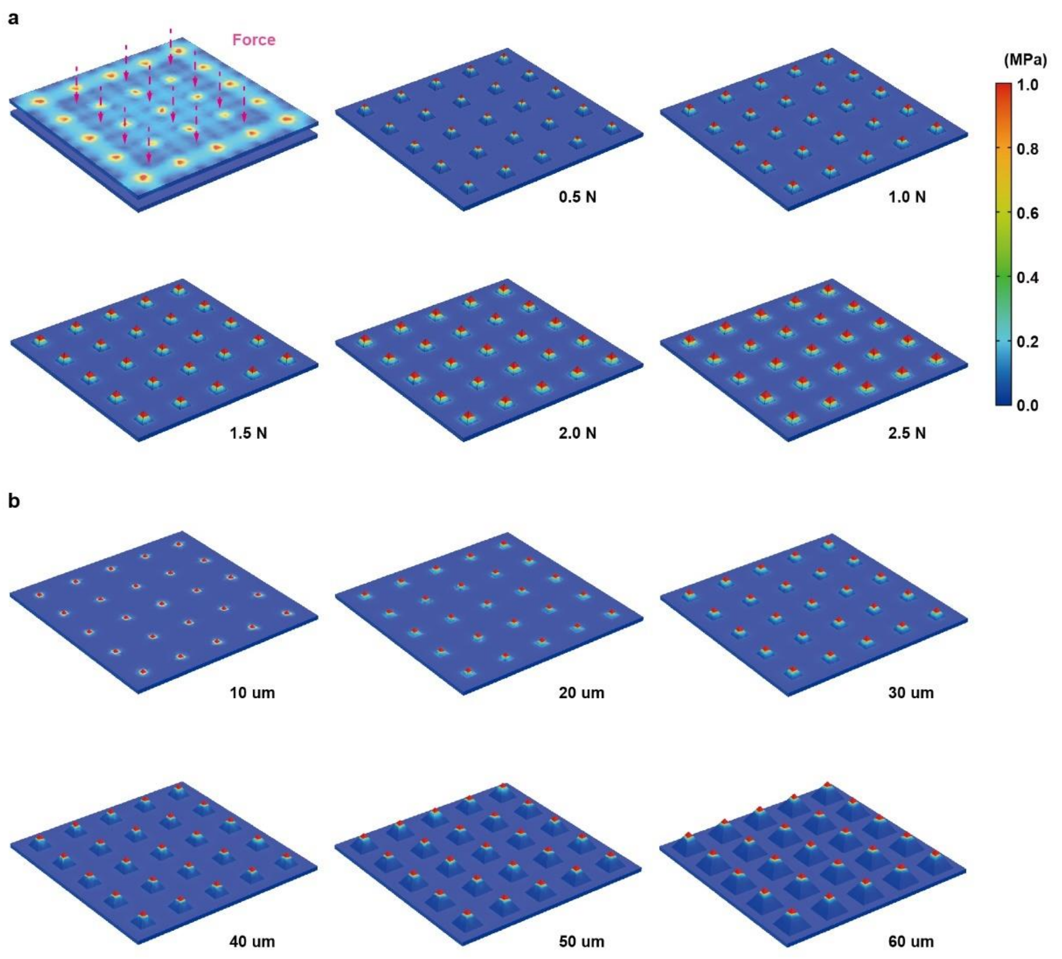

Supplementary Figure 12 | Simulation analysis of microstructured PDMS pyramid array under external force. The stress distribution of simulation results for microstructured PDMS with the force (**a**) ranging from 0.5 N to 2.5 N and the pyramid size (**b**) ranging from 10 um to 60 um.

Supplementary Figure 12a shows the stress distribution of the pressure sensor with the pyramid size of 30 um under different forces. It could be found that the stress distribution of the pyramid gradually increases with external force, indicating that the contact between two layers is getting closer. Meanwhile, the stress distribution of sensors with different pyramid sizes under 1 N force is illustrated in Supplementary Fig. 12b. For the smaller pyramids, the whole pyramid structure and even the bottom layer show the stress concentration, suggesting that the top and bottom electrodes are in close contact. Hence, this type of sensor usually possesses a higher sensitivity, but its pressure detection range is relatively narrow.

Supplementary Note 5: Principle and performance of pressure sensor

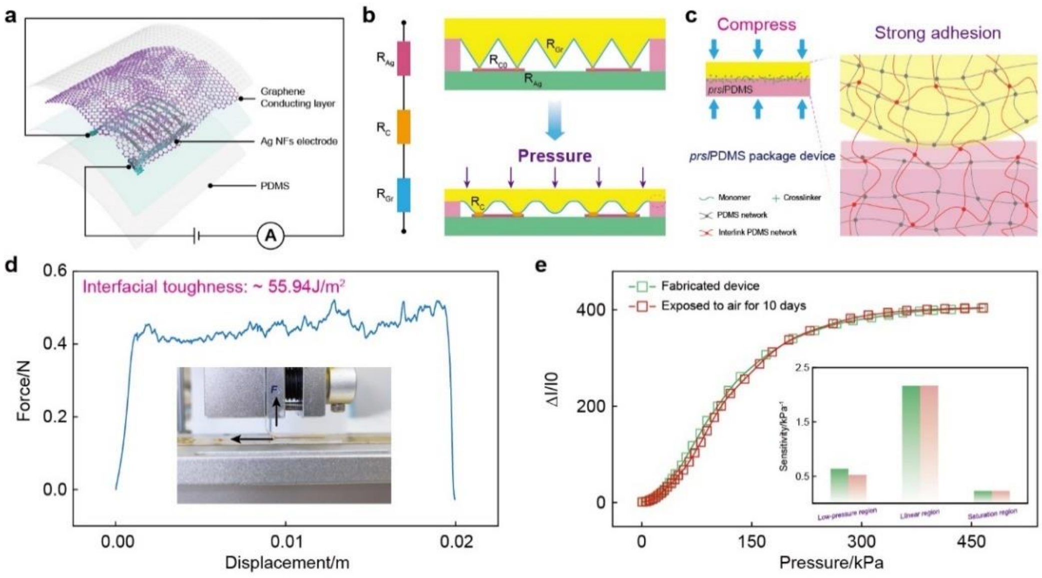

Supplementary Figure 13 | The working principle and stability of the device. (**a**) Schematic illustration of the measurement setup. (**b**) Working principle of the pressure sensor. (**c**) Principle of robust interfacial adhesion between the top and bottom electrodes. (**d**) Characterization of interfacial toughness of device. (**e**) Performance comparison of device exposed to air for 10 days. Inset: Sensitivity comparison of devices in different regions.

The pressure sensor is composed of microstructured graphene layer, prslPDMS layer and Ag NFs interdigital electrodes, as shown in Supplementary Fig. 13a. A circuit model has been proposed to analyze the resistance variation of the sensor under external pressure (Supplementary Fig. 13b). It could be found that the resistance of the sensor is affected by three aspects, including the resistance of graphene (R_{Gr}), the resistance of Ag NFs (R_{Ag}) and the contact resistance between two electrodes (R_C). Hence, the current of sensor could be calculated by the following equation:

$$I = U / (R_{Ag} + R_C + R_{Gr}) \quad (5\text{-}1)$$

The R_{Ag} and R_{Gr} are the constant value which could be regulated by improving the preparation process. Therefore, the contact resistance (R_C) is the main factor affecting the conductance of the sensor, which mainly depends on the contact area. The small contact area at a low pressure shows a large contact resistance, and the contact area

increasing rapidly with the external pressure, leading to an increase in conductivity of the sensor. Supplementary Figure 13c describes the principle of interfacial adhesion between the top and bottom layers. The prslPDMS layer is swollen in toluene during its preparation. After stacking the swollen layer with the microstructure layer and continuously applying certain pressure, monomers and crosslinkers will infiltrate into the preformed PDMS networks. Then, a new crosslinking network is formed in topological entanglement with preformed polymer networks, thus tightly adhering the two different layers. The interfacial toughness was measured about 55.94 J/m^2, indicating that the electrode interfaces achieve the sufficient adhesion and can isolate external interference (Supplementary Fig. 13d). Therefore, the device shows an excellent performance, and its pressure detection ability is similar to the initial state even after exposure to ambient environment for 10 days (Supplementary Fig. 13e).

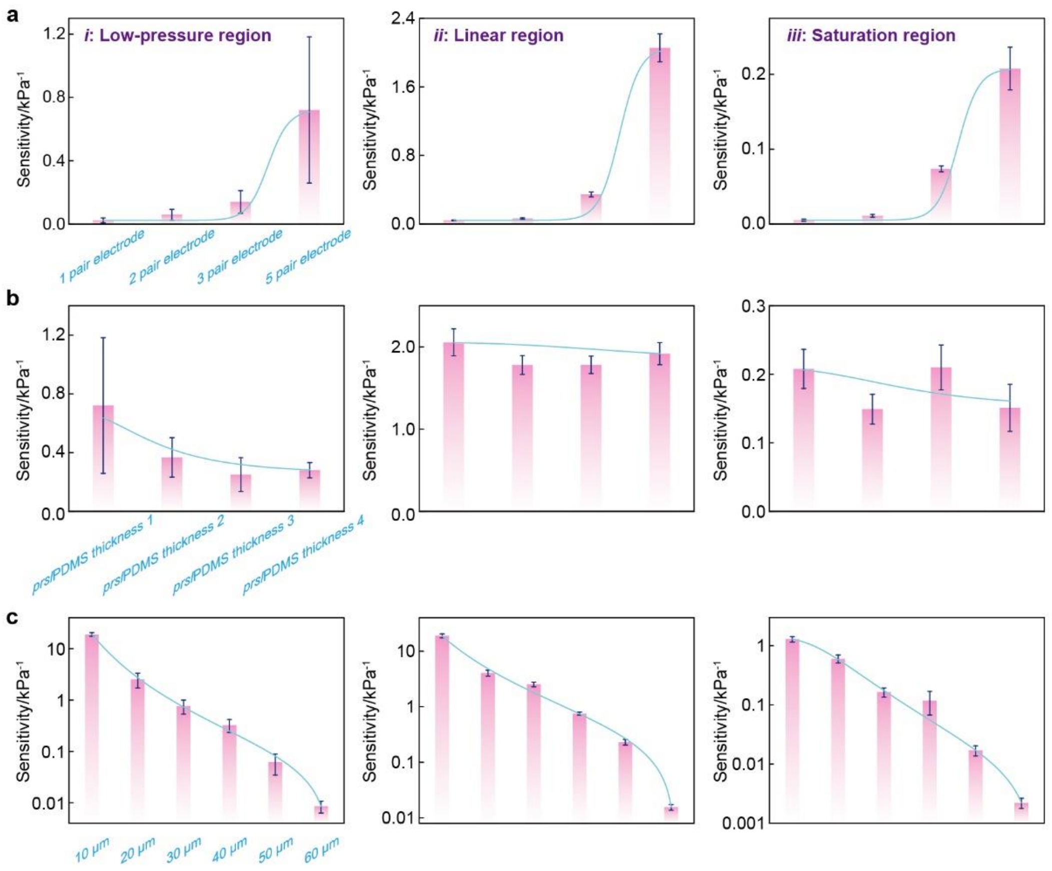

Supplementary Figure 14 | Sensitivity analysis of the sensors with different interdigital electrodes (a), spacer layer thickness (b) and pyramid sizes(c).

Extensive data show that the sensitivity curve of the pressure sensor in this work could be divided into three regions, low-pressure region, linear region, and saturation region. The denser interdigital electrodes would significantly improve the sensor sensitivity. Since more electrodes could provide more contact sites, resulting in the dramatic current variation. For example, the sensitivity changes from 0.042 kPa^{-1} to 2.06 kPa^{-1} in the linear region. Nevertheless, the thickness of the prslPDMS layer has the little effect on its sensitivity, and its value in the linear region is about 1.88 kPa^{-1}. For the upper layer with different pyramid size, larger pyramid means the lower sensitivity, which declines from 18.94 kPa^{-1} to 0.015 kPa^{-1} in linear region. This could be attributed to the fact that smaller pyramid enables the two layers in close contact under less force, resulting in a more sensitive current variation, which is consistent with the previous simulation results.

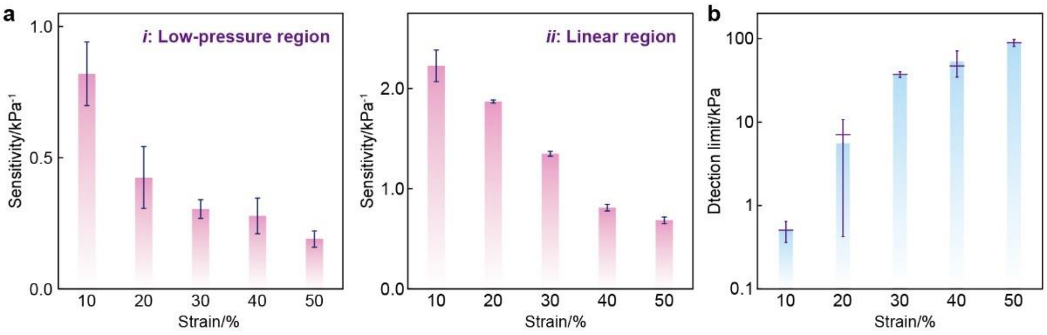

Supplementary Figure 15 | Sensitivity (a) and detection limit (b) of sensors under different deformations.

It could be seen that the sensitivity of the sensor gradually decreases with the larger strain. Taking the linear region as an example, the sensitivity changes from 2.23 kPa^{-1} to 0.68 kPa^{-1} when the strain increases to 50%. Additionally, the lower detection limit has also increased significantly, from ~500 Pa to ~90 kPa. Tensile strain will destroy the integrity of the interdigital Ag NFs or Gr electrode, so more loops with good conduction are formed only after more pressure is applied, leading to the decreased sensitivity and lower detection limits. However, its tactile detection performance could return to the original state when the strain is released.

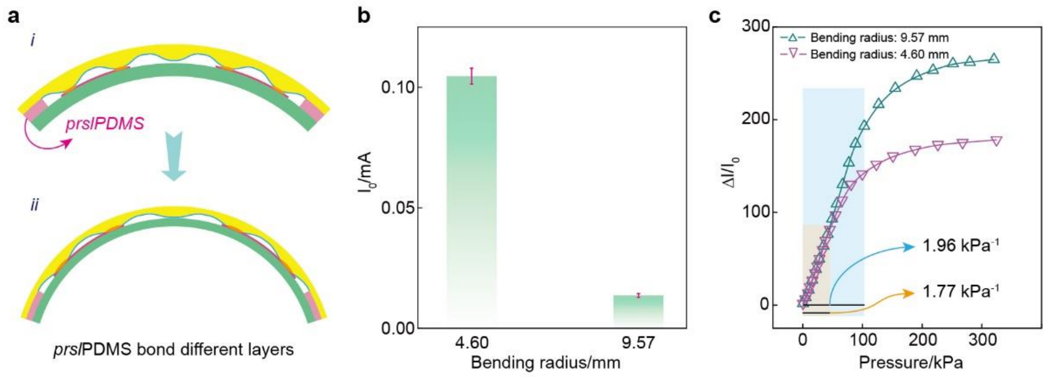

Supplementary Figure 16 | Pressure response performance of the sensor under bending condition.

Supplementary Figure 16a describes the schematic diagram of the device under bending condition, in which prslPDMS layer has a good adhesion effect on the upper and bottom layers, so there is still an air gap in sensor. However, the air gap decreased with the smaller bending radius, which also means the sensor is in the linear response region. The currents in two different bending states are illustrated in Supplementary Fig. 16b, and the smaller radius shows the larger current, indicating a closer contact between electrodes. The pressure response performance of the sensor under bending conditions was further explored. The results show that its sensitivity in the linear region is basically the same, but the device with smaller bending radius presents lesser pressure detection range, which quickly enters the saturation region. For the sensor with bending radius of 9.57 mm, the linear pressure response range extends to 100 kPa (Supplementary Fig. 16c), and this result is consistent with theoretical analysis.

Supplementary Note 6: Pulse wave monitoring with ultrathin sensor

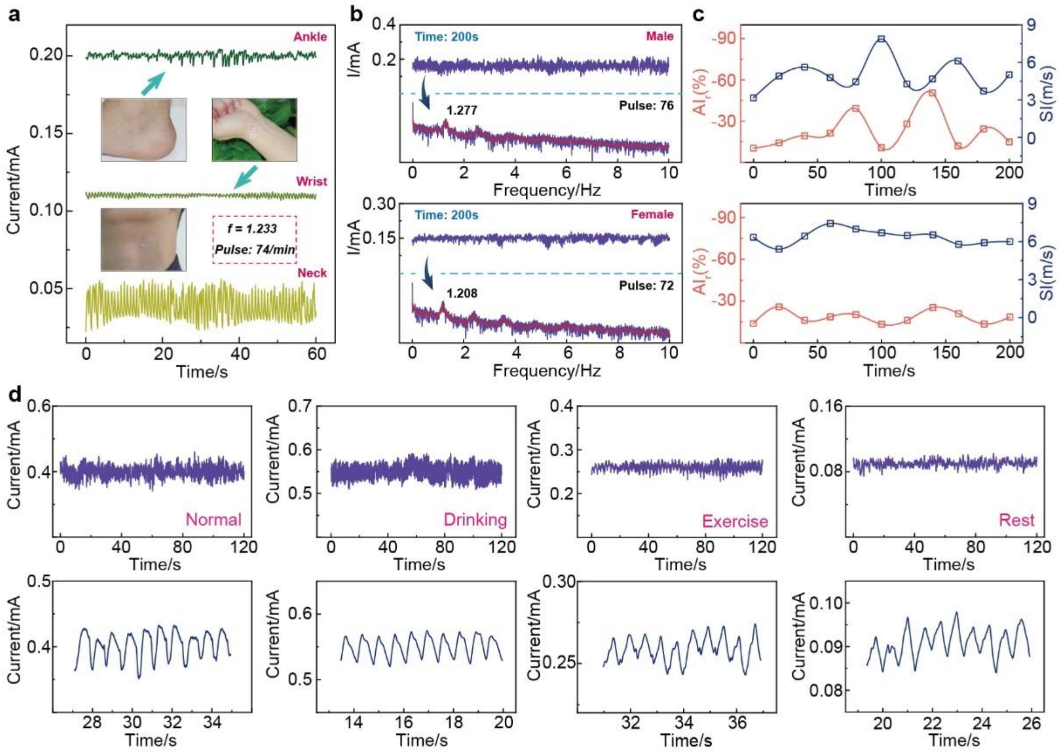

Supplementary Figure 17| Detection and analysis of the human pulse. (**a**) Human pulse measurement at the ankle, wrist and neck, respectively. (**b**) Long-term pulse monitoring test for the 25-year-old male and female. The frequency spectrums of the pulse wave through Fourier transform are shown below. (**c**) Comparison of the *RI* value and *AIx* value between the male and female. (**d**) Real-time pulse measurement and analysis of human body under different conditions.

Supplementary Figure 17a shows the arterial pulse obtained from the skin surface, such as the neck, wrist, and ankle. The pulse signals are relatively stronger at the neck than wrist or ankle, so the current on the carotid artery has the small baseline and large current variation. The ultrathin sensor could accurately detect the pulse at the ankle with the frequency of 1.233 Hz, and the heart rate could be calculated as about 74/min. Long-term pulse detection is still vital for human in daily life, and a time of 200 s is used to continuously record the arterial pulse of 25-year-old male and female (Supplementary Fig. 17b). In order to acquire more sufficient pulse information, the corresponding frequency spectrums obtained by Fourier transform are displayed below. It could be found that the significant peaks for man and woman are about 1.277 and 1.208 Hz,

respectively, and other frequency peaks are concentrated below 5 Hz, indicating that the arterial pulse belongs to the low-frequency signal. The corresponding augmentation index (*AIx*) and reflection index (*RI*) are shown in the Supplementary Fig. 17c, which are calculated by selecting a set of data every 10 seconds. The results show that the indexes of male fluctuate significantly compared with that of female, indicating that the pulse of male is less stable. Moreover, Supplementary Fig. 17d illustrates the pulse variation of human body under different conditions. Obviously, the heart rate will increase after drinking or exercising, and we believe these conclusions will help the medics to analyze the changes of the cardiovascular system and provide better health monitoring.

Supplementary Note 7: Multiple sensors for grip detection

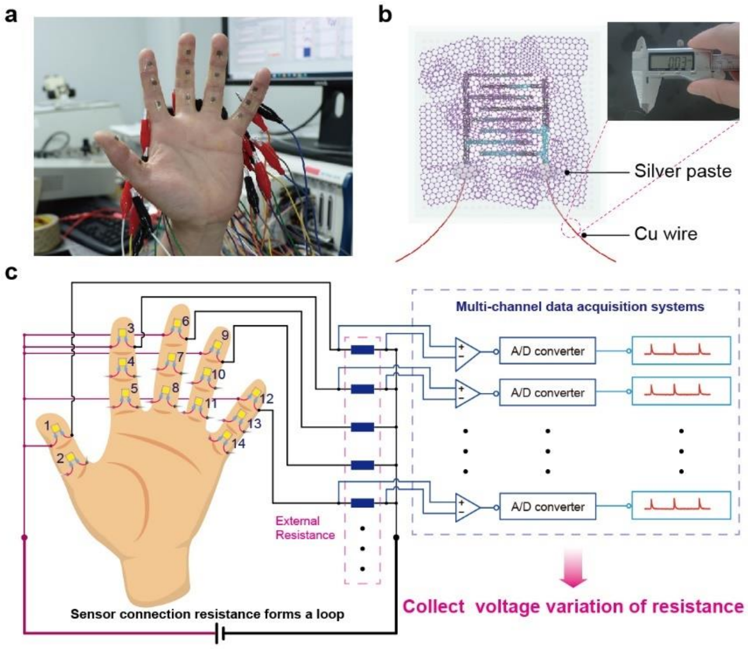

Supplementary Figure 18 | Measurement principle with multiple sensors attached to the palm.

Multiple sensors attached to the palm is described in Supplementary Fig. 18a. Each sensor is fixed with scotch tape, then connected in series to the loop with alligator clips. The sensor in this work is very thin, so the fine copper wires (~ 30 μm) are used to lead out the electrodes, and silver paste is used to connect the interdigital electrodes and wires. Additionally, use PDMS to encapsulate the silver paste to prevent it from peeling off (Supplementary Fig. 18b). Supplementary Figure 18c demonstrates the principle of simultaneous measurement of multiple sensors. Since the data acquisition card (PXIe-4300, National Instruments) in the lab could only collect the voltage, we connect an external resistor in series with each sensor. By measuring the voltage of the resistance, the current flowing through it could be calculated. According to the Kirchhoff's law, the current in the entire loop is equal everywhere, so this current could be considered as the current flowing through the sensor. Therefore, 14 sensors need 14 external resistors, and 14 loops are connected in parallel with each other.

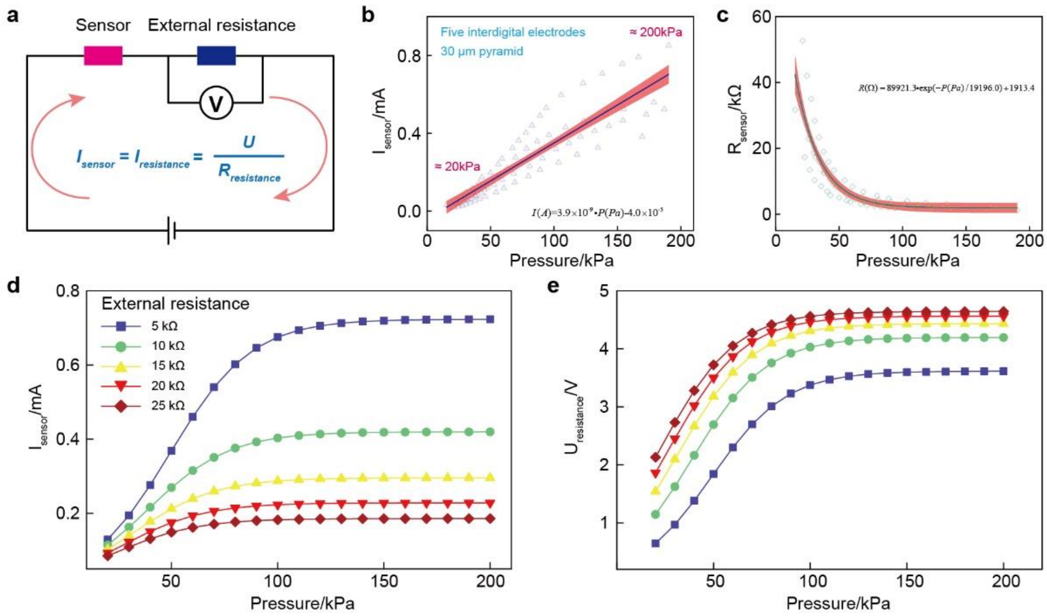

Supplementary Figure 19 | The conversion principle of current and voltage in multi-channel data acquisition. (**a**) Equivalent circuit diagram of single-channel signal acquisition. Fitted curves of current (**b**) and resistance (**c**) of the sensor in the linear region of 20 to 200 kPa. Current variation (**d**) and the voltage variation on external resistance (**e**) with different resistors in series to the loop.

Supplementary Figure 19a depicts the equivalent circuit of single-channel signal acquisition. Based on the Kirchhoff's law, the current of the sensor could be obtained by the following formula:

$$I_s = I_r = \frac{U_r}{R_r} \tag{7-1}$$

I_s: the current of pressure sensor, I_r: the current of external resistance, U_r: the voltage of external resistance; Furthermore, the voltage of external resistance could also be deduced as follows:

$$U_r = \frac{R_r}{R_s + R_r} U_s \tag{7-2}$$

U_s: the voltage of power supply;

According to previous measurement data, the empirical formula of the current on sensor with the pressure could be fitted (Supplementary Fig. 19b), then the relationship between its resistance and pressure could also be calculated (Supplementary Fig. 19c):

$$I_s = 3.9\times10^{-9}\bullet P - 4.0\times10^{-5} \tag{7-3}$$

$$R_s = 89921.3\bullet\exp(\frac{-P}{19196.0}) + 1913.4 \tag{7-4}$$

Subsequently, the influence of different external resistors on loop current and measurement voltage U_r is further studied. It could be found that the loop current gradually declines with the larger external resistance, but the range of U_r increases slightly which is beneficial for signal acquisition.

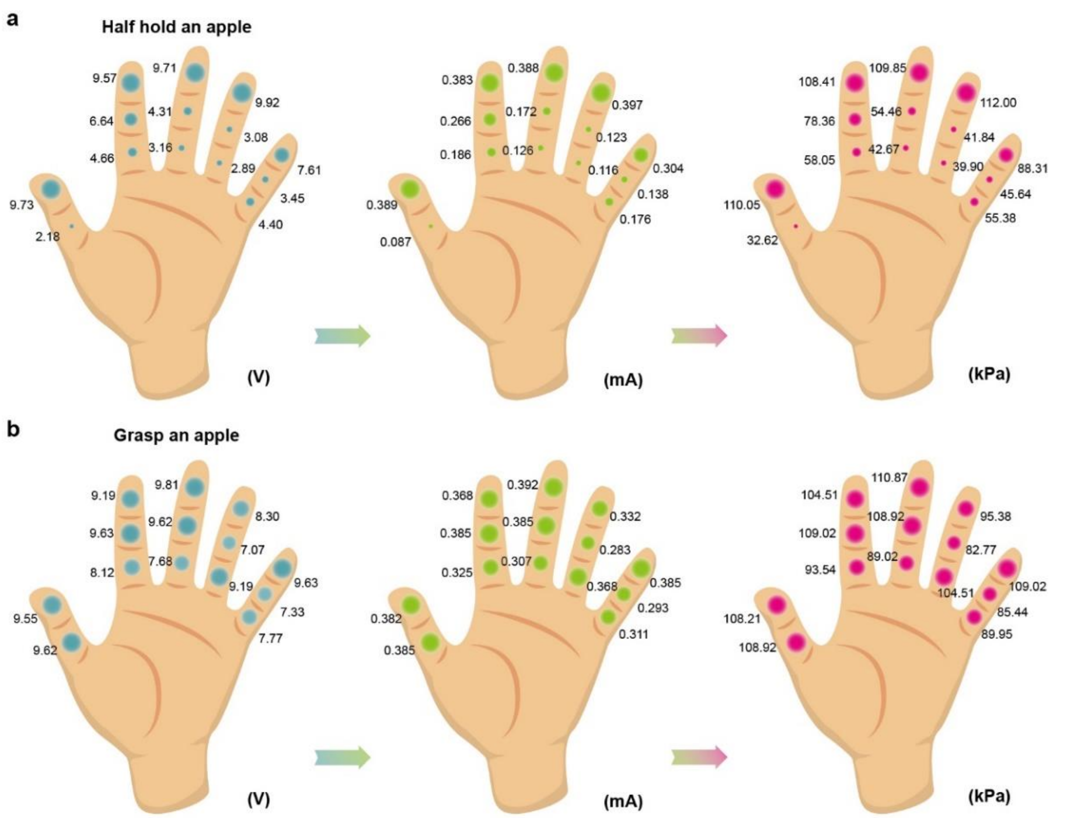

Supplementary Figure 20 | The current and pressure signal diagram of multiple sensors in different grip postures.

The power supply used in this experiment is 10 V, and the external resistance is 25 kΩ. According to the above theoretical analysis, the pressure and current of the sensor could be calculated by the following formulas:

$$I_s = \frac{U_r}{25000} \tag{7-5}$$

$$U_r = \frac{250000}{89921.3 \bullet \exp(\frac{-P}{1919.6}) + 26913.4} \tag{7-6}$$

It could be found that the current and pressure both show the same variation trend, which is consistent with actual situation, so we choose the voltage (U_r) to reflect the real-time pressure distribution.

Supplementary Note 8: Principle and measurement system for sensor arrays

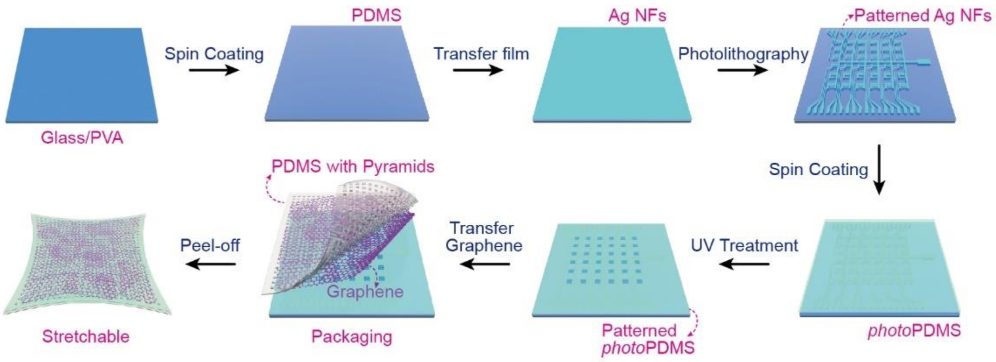

Supplementary Figure 21 | Schematic diagram of the preparation process for array device.

The whole preparation process for array device is schematically shown in the Supplementary Fig. 21, which is summarized below:

1. The polyvinyl alcohol (PVA) water solution was spin-coated on the glass substrate followed by baking at 100 °C for 120s. The PDMS was then spin-coated on the PVA/glass substrate at high rotation speed as an ultrathin elastic substrate.

2. The bottom Ag NFs film was transferred on the PDMS substrate, and the patterned Ag NFs electrodes were fabricated by a series of micromachining technology, details could be found in Supplementary Note 2.

3. The patterned prslPDMS was prepared on the Ag NFs electrodes as the spacer layer, the specific preparation steps could refer to Supplementary Note 1. The prslPDMS layer is essential to the sensor, which can not only expose the interdigital electrodes and encapsulate the transmission, but also form the micro-cage structure that greatly reduces mechanical crosstalk.

4. The microstructured PDMS layer was prepared through the silicon template, then an interfacially self-assembled graphene film was transferred on its surface. The PDMS/Gr layer will be laminated on the prslPDMS layer for sensor assembly.

5. Finally, the sensor could be peeled off by dissolving the PVA sacrificial layer in water.

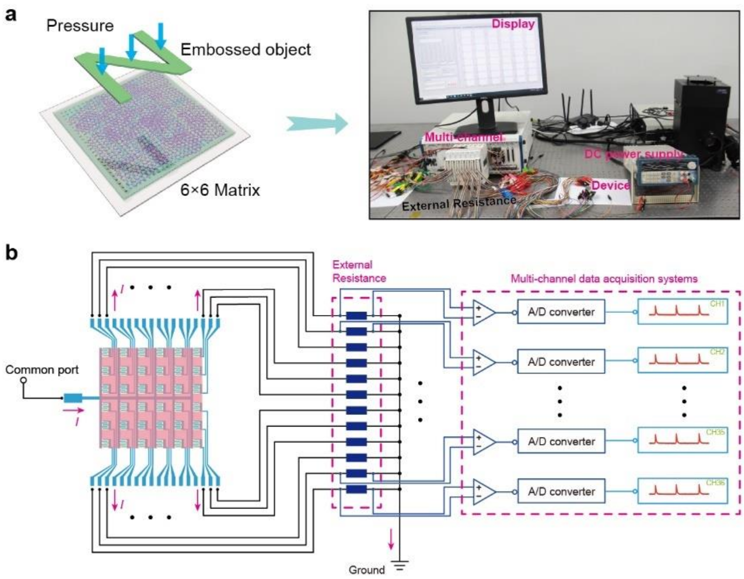

Supplementary Figure 22 | Multi-channel data measurement system. (**a**) Schematic illustration of the pressure mapping process (left). Optical image of the data measurement system (right). (**b**) The principle of the multi-channel synchronous data acquisition.

Supplementary Figure 22a demonstrates the schematic diagram of a 6 × 6 sensor array for pressure detection. The electrical signal of the pixels will change with the objects placed on its surface, thereby real-time presenting the shape of the object. The measurement principle here is similar to that of the previous multiple sensors, and each pixel in the array is connected to a resistor. Multi-channel synchronous data acquisition card collects the voltage on the resistor, then calculates the pressure on the pixel, which is equivalent to multi-electrometer scanning simultaneously.

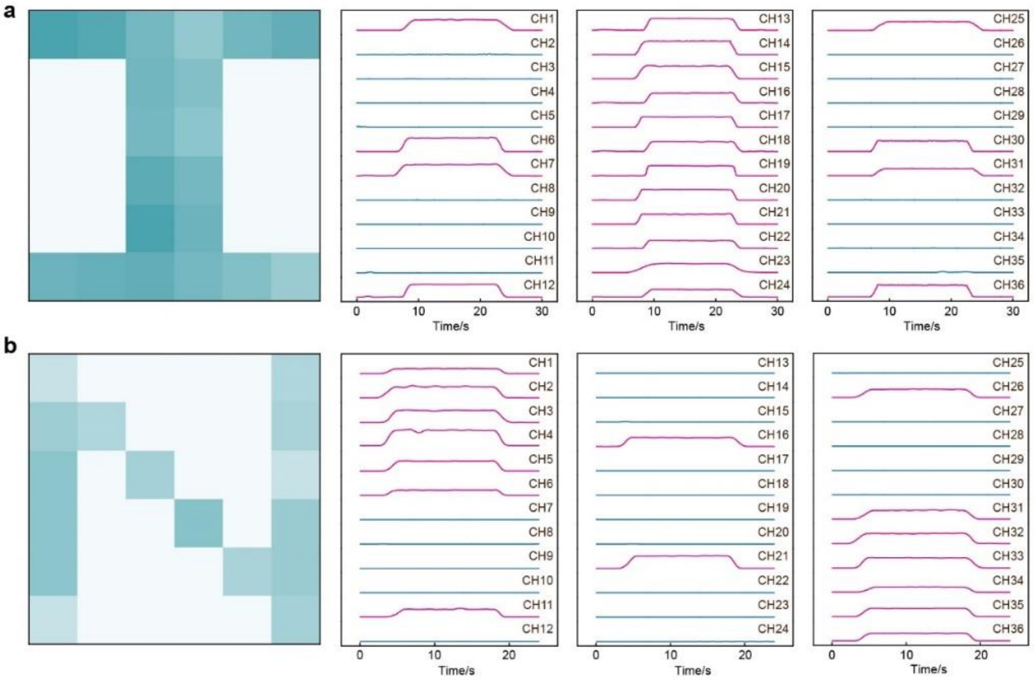

Supplementary Figure 23 | Pressure imaging and voltage signal measured by using the synchronous data acquisition card system.

Supplementary Figure 23 exhibits the voltage variation of the sensor array by pressing a self-made embossed object (letter "I" and "N") on its surface. The results show that voltage of pixels under pressure increases significantly, while the voltage of other pixels has almost no change.

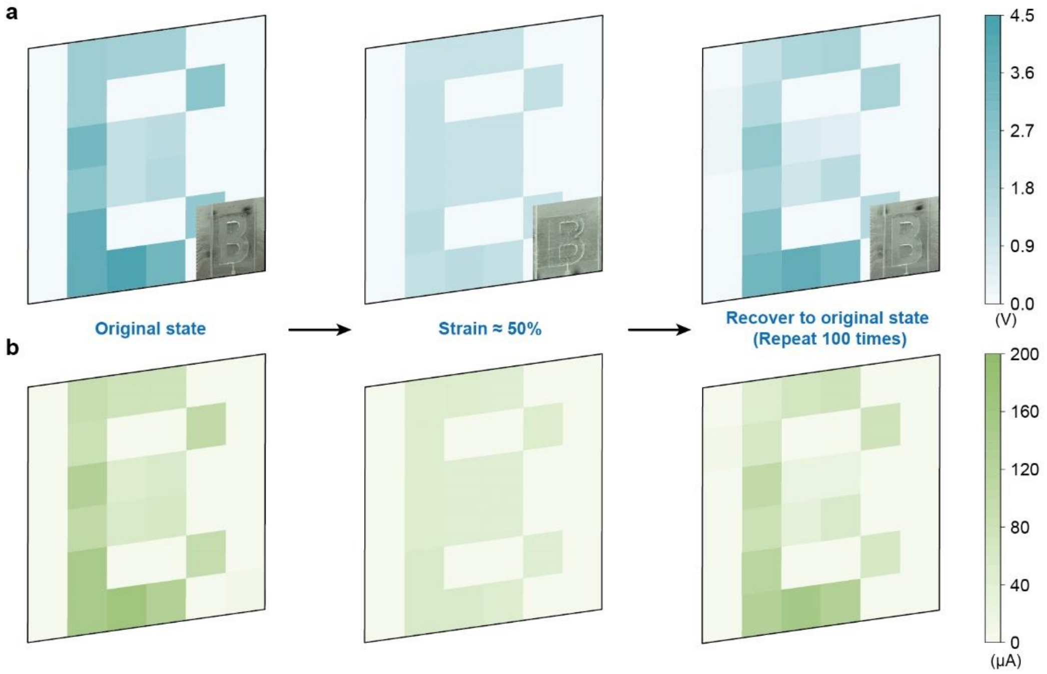

Supplementary Figure 24 | Imaging performance of sensor array under tensile strain.

When the device is subjected to 50% tensile strain, the voltage measured at each pixel drops significantly, which means that the stretching will increase the resistance of each pixel. Although the tactile sensing performance has decreased, the pattern of the letter "B" could still be observed. Furthermore, when the deformation is released after 100 times of cyclic stretching, its performance roughly returns to the original state, indicating the good durability. The current variation shown below is also consistent with the above results, indicating that the sensor array realizes an excellent stability.

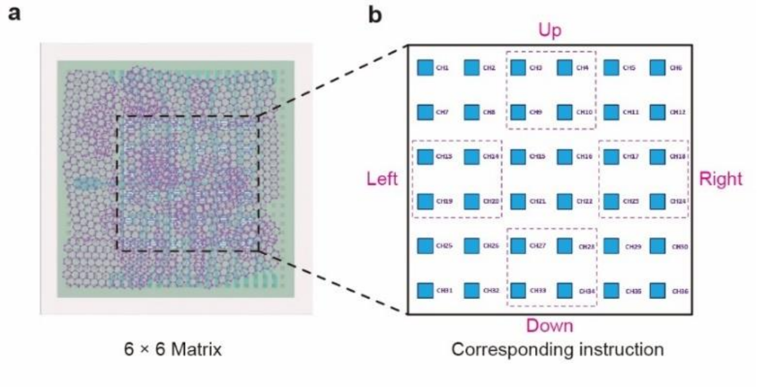

Supplementary Figure 25 | Schematic diagram of the instruction distribution of the array deivce.

The pixels of the 6 × 6 pressure sensor array were divided into four regions, including moving up, down, left and right, as shown in Supplementary Fig. 25. Once an object contacts with the sensor, the corresponding voltage variation will be recorded by the multi-channel data acquisition card. After analyzing by the customized LabVIEW program, a move order is then transmitted to the model in the game.

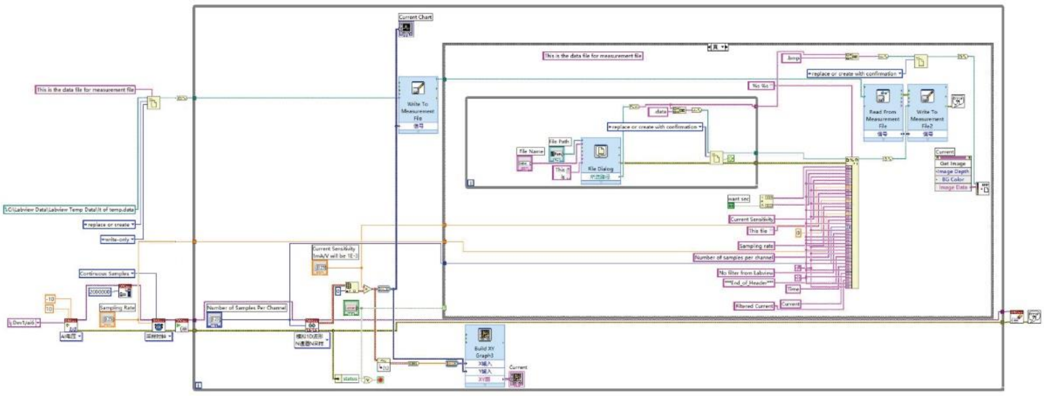

Supplementary Figure 26 | LabVIEW program for single sensor to collect current.

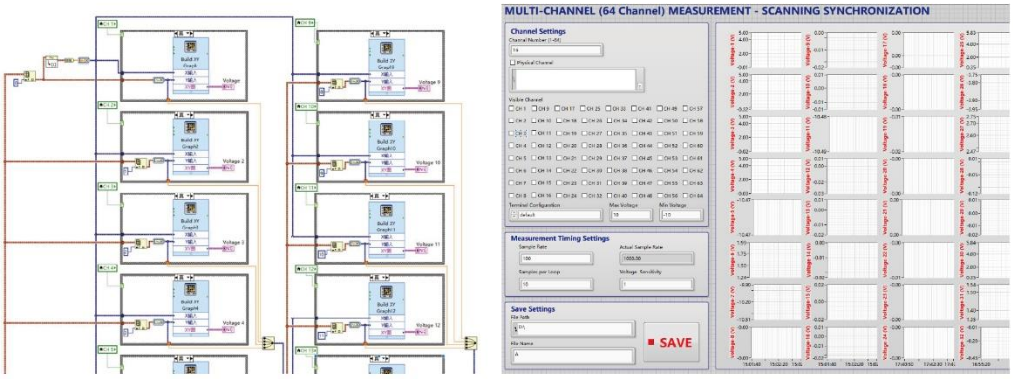

Supplementary Figure 27 | LabVIEW program for multi-channel synchronous voltage acquisition.

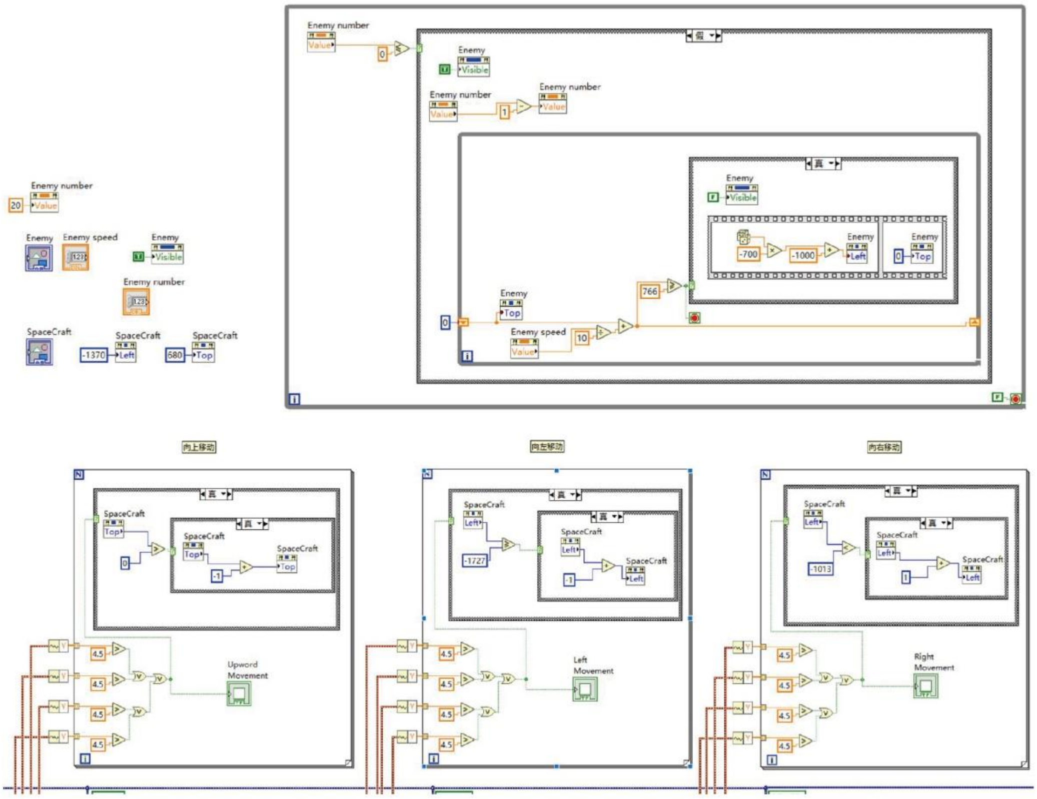

Supplementary Figure 28 | LabVIEW program for human-machine interaction game.

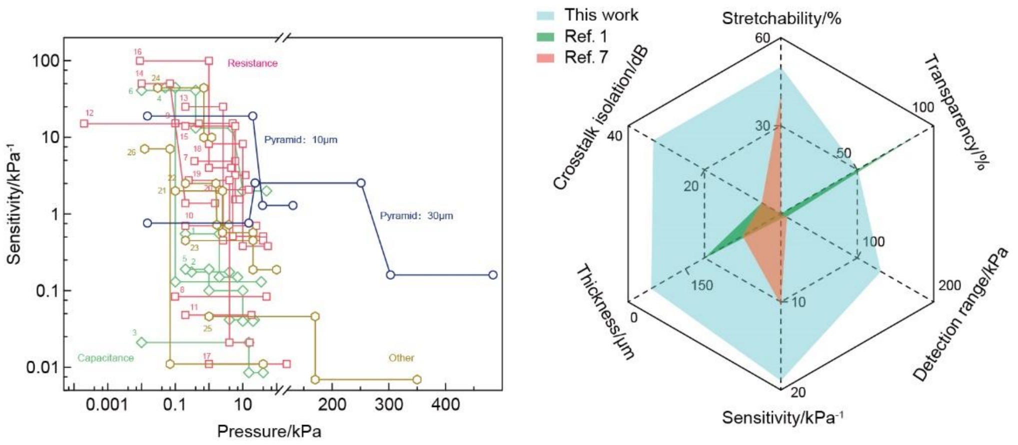

Supplementary Figure 29 | Performance comparison with state-of-the-art pressure sensors.

Comparison of our sensor with existing sensors in terms of sensitivity and detection range are depicted in Supplementary Fig. 29[1-26]. Although it is not the most sensitive, it shows a wider linear response range. The two representative works are selected to further analyze the advantages of our sensor (shown on right), including stretchability, thickness, sensitivity, transparency, detection range and crosstalk isolation. It could be seen that our sensor has good performance in all aspects, which can be attached to human skin with large scale for precise tensile detection.

Supplementary References

1. Mannsfeld S. C. B., et al. Highly sensitive flexible pressure sensors with microstructured rubber dielectric layers. *Nat. Mater.* **9**, 859-864 (2010).
2. Tee B. C. K., Chortos A., Dunn R. R., Schwartz G., Eason E., Bao Z. A. Tunable Flexible Pressure Sensors using Microstructured Elastomer Geometries for Intuitive Electronics. *Adv. Funct. Mater.* **24**, 5427-5434 (2014).
3. Ruth S. R. A., Beker L., Tran H., Feig V. R., Matsuhisa N., Bao Z. A. Rational Design of Capacitive Pressure Sensors Based on Pyramidal Microstructures for Specialized Monitoring of Biosignals. *Adv. Funct. Mater.* **30**, 1903100 (2020).
4. Yang J. C., et al. Microstructured Porous Pyramid-Based Ultrahigh Sensitive Pressure Sensor Insensitive to Strain and Temperature. *Acs Appl. Mater. Inter.* **11**, 19472-19480 (2019).
5. Boutry C. M., et al. A hierarchically patterned, bioinspired e-skin able to detect the direction of applied pressure for robotics. *Sci. Robot.* **3**, eaau6914 (2018).
6. Cho S. H., et al. Micropatterned Pyramidal Ionic Gels for Sensing Broad-Range Pressures with High Sensitivity. *Acs Appl. Mater. Inter.* **9**, 10128-10135 (2017).
7. Choong C. L., et al. Highly Stretchable Resistive Pressure Sensors Using a Conductive Elastomeric Composite on a Micropyramid Array. *Adv. Mater.* **26**, 3451-3458 (2014).
8. Gao Yuji, et al. Wearable Microfluidic Diaphragm Pressure Sensor for Health and Tactile Touch Monitoring. *Adv. Mater.* **29**, 1701985 (2017).
9. Wang Y., Wu H. T., Xu L., Zhang H. N., Yang Y., Wang Z. L. Hierarchically patterned self-powered sensors for multifunctional tactile sensing. *Sci. Adv.* **6**, eabb9083 (2020).
10. Bae G. Y., et al. Pressure/Temperature Sensing Bimodal Electronic Skin with Stimulus Discriminability and Linear Sensitivity. *Adv. Mater.* **30**, 1803388 (2018).
11. Matsuda Ryosuke, et al. Highly stretchable sensing array for independent detection of pressure and strain exploiting structural and resistive control. *Sci. Rep.* **10**, 12666 (2020).

12. Park J., et al. Giant Tunneling Piezoresistance of Composite Elastomers with Interlocked Microdome Arrays for Ultrasensitive and Multimodal Electronic Skins. *Acs Nano* **8**, 4689-4697 (2014).

13. Pang Y., et al. Epidermis Microstructure Inspired Graphene Pressure Sensor with Random Distributed Spinosum for High Sensitivity and Large Linearity. *Acs Nano* **12**, 2346-2354 (2018).

14. Su B., Gong S., Ma Z., Yap L. W., Cheng W. L. Mimosa-Inspired Design of a Flexible Pressure Sensor with Touch Sensitivity. *Small* **11**, 1886-1891 (2015).

15. Bae Geun Yeol, et al. Linearly and Highly Pressure-Sensitive Electronic Skin Based on a Bioinspired Hierarchical Structural Array. *Adv. Mater.* **28**, 5300-5306 (2016).

16. Gao Yuyu, et al. Microchannel-Confined MXene Based Flexible Piezoresistive Multifunctional Micro-Force Sensor. *Adv. Funct. Mater.* **30**, 1909603 (2020).

17. Park Y. J., et al. All MoS2-Based Large Area, Skin-Attachable Active-Matrix Tactile Sensor. *Acs Nano* **13**, 3023-3030 (2019).

18. Schwartz G., et al. Flexible polymer transistors with high pressure sensitivity for application in electronic skin and health monitoring. *Nat. Commun.* **4**, 1859 (2013).

19. Takahashi T., Takei K., Gillies A. G., Fearing R. S., Javey A. Carbon Nanotube Active-Matrix Backplanes for Conformal Electronics and Sensors. *Nano Lett.* **11**, 5408-5413 (2011).

20. Takei K., et al. Nanowire active-matrix circuitry for low-voltage macroscale artificial skin. *Nat. Mater.* **9**, 821-826 (2010).

21. Zhu Pengcheng, Wang Yalong, Wang Yao, Mao Hongye, Zhang Qiang, Deng Yuan. Flexible 3D Architectured Piezo/Thermoelectric Bimodal Tactile Sensor Array for E-Skin Application. *Adv. Energy Mater.* **10**, 2001945 (2020).

22. Rao J. H., et al. Tactile electronic skin to simultaneously detect and distinguish between temperature and pressure based on a triboelectric nanogenerator. *Nano Energy* **75**, 105073 (2020).

23. Ha M., et al. Skin-Inspired Hierarchical Polymer Architectures with Gradient Stiffness for Spacer-Free, Ultrathin, and Highly Sensitive Triboelectric Sensors. *Acs Nano* **12**, 3964-3974 (2018).

24. Meng K. Y., et al. Flexible Weaving Constructed Self-Powered Pressure Sensor Enabling Continuous Diagnosis of Cardiovascular Disease and Measurement of Cuffless Blood Pressure. *Adv. Funct. Mater.* **29**, 1806388 (2019).
25. Li Z. L., Zhu M. M., Shen J. L., Qiu Q., Yu J. Y., Ding B. All-Fiber Structured Electronic Skin with High Elasticity and Breathability. *Adv. Funct. Mater.* **30**, 1908411 (2020).
26. Peng X., et al. A breathable, biodegradable, antibacterial, and self-powered electronic skin based on all-nanofiber triboelectric nanogenerators. *Sci. Adv.* **6**, eaba9624 (2020).
