# Supplementary material for: Localizing strain via micro-cage structure for stretchable pressure sensor arrays with ultralow spatial crosstalk
Source: Nat Commun. 2023 Mar 6;14:1252. doi: 10.1038/s41467-023-36885-3 (PMC9988987; doi:10.1038/s41467-023-36885-3)
Supplement: Supplementary file 3 — Description of Additional Supplementary Files [file 41467_2023_36885_MOESM3_ESM.docx]

Supplementary movie 1

Description: Electrospinning and graphene preparation;

Supplementary movie 2

Description: Pulse detection under different pressures;

Supplementary movie 3

Description: Multiple sensors used for grasping posture analysis;

Supplementary movie 4

Description: Demo of application scenarios;
